# Evaluation the Risk of Preeclampsia in Periodontal Patients Compared With Healthy Individuals: A Meta-Analysis Study

**DOI:** 10.1155/ijod/3360082

**Published:** 2025-06-03

**Authors:** Somayeh Ansari Moghaddam, Shiva Shayesteh, Alireza Ranjbaran

**Affiliations:** ^1^Department of Periodontology, School of Dentistry, Zahedan University of Medical Sciences, Zahedan, Iran; ^2^Department of Periodontology, Faculty of Dentistry, Hamedan University of Medical Sciences, Hamedan, Iran

**Keywords:** meta-analysis, periodontitis, pre-eclampsia

## Abstract

Periodontal disease (PD) is a common oral condition that has been suggested to have potential adverse effects on pregnancy outcomes, including preeclampsia. This meta-analysis aims to investigate the association between PD and preeclampsia. A comprehensive search of relevant studies was conducted, and data were analyzed using a random effects model with the Mantel–Haenszel method to estimate the pooled odds ratio (OR) and assess heterogeneity. The results revealed that women with PD had a significantly higher risk of developing preeclampsia (OR = 1.20, 95% CI: 1.01–1.42). Heterogeneity among the studies was minimal (*I*^2^ = 43.8%), despite the chi-squared test indicating some heterogeneity (*χ*^2^ = 48.03, d.f. = 27, *p*=0.008). These findings underscore the importance of incorporating periodontal care into prenatal care routines to mitigate the risk of adverse pregnancy outcomes.

## 1. Introduction

Periodontal diseases (PDs) are among the most common chronic infectious diseases in humans, affecting the soft and hard tissues surrounding the teeth [1, 2]. The primary causative agents of PD are dental plaque and calculus [2]. Given its inflammatory nature, researchers have proposed that PD might potentially influence the development of some systemic diseases [3–6].

In the United States, at least 35% of adults aged 30–90 experience PD, and it may affect up to 90% of the global population [7]. Previous studies have shown that PD can impact atherosclerotic cardiovascular disease, diabetes, adverse pregnancy outcomes, chronic obstructive pulmonary disease (COPD), osteoporosis, and rheumatoid arthritis [3–6, 8]. One of the diseases affected by PD is preeclampsia [9].

Preeclampsia is a maternal syndrome characterized by proteinuria and hypertension, affecting both the mother and fetus [9]. It involves, on average, 8.5% of pregnant women and is the second most common cause of maternal mortality worldwide [9]. Women with chronic low-grade inflammatory conditions, such as diabetes mellitus, hypertension, obesity, and arterial diseases, are at increased risk of developing preeclampsia. Given that PD is also associated with low-grade inflammation, it is hypothesized that patients with PD may have an increased risk of developing preeclampsia.

The biological mechanisms linking PD and preeclampsia are not yet fully understood but are believed to involve systemic inflammation and bacterial translocation. Periodontal infection triggers a persistent inflammatory response characterized by elevated levels of pro-inflammatory cytokines, such as interleukin-6 (IL-6), tumor necrosis factor-alpha (TNF-*α*), and C-reactive protein (CRP) [10]. These inflammatory mediators may enter the bloodstream, contributing to endothelial dysfunction and placental inflammation, both of which play a crucial role in the pathogenesis of preeclampsia [9]. Additionally, periodontal pathogens like *Porphyromonas gingivalis* can translocate to the placenta, potentially exacerbating local inflammation and further increasing the risk of preeclampsia [9].

Meta-analysis studies on the association between PD and preeclampsia by Wei et al. [10] in 2013 and Huang et al. [11] in 2014 indicated a connection between the two conditions. While most recent studies also suggest an association between PD and preeclampsia, there are still studies indicating no significant relationship between the two [2]. Thus, the potential role of PD in the pathogenesis of preeclampsia remains an important but unresolved issue.

A comprehensive systematic review and meta-analysis of studies, incorporating recent findings on the association between PD and preeclampsia, can provide more precise and scientific evidence for clinicians, researchers, and policymakers. This study aimed to conduct a systematic review and meta-analysis to evaluate the risk of preeclampsia in periodontal patients compared to healthy individuals.

## 2. Methods

### 2.1. Study Population

This meta-analysis in 2021 comprised all articles published in the databases Scopus, Web of Science, PubMed, Science Direct, Cochrane Library, and the Google Scholar search engine regarding PD and preeclampsia during pregnancy, where blood pressure, urine protein levels, and risk levels were reported.

#### 2.1.1. Inclusion and Exclusion Criteria

The inclusion and exclusion criteria for this meta-analysis are summarized in Table 1. To ensure the consistency and reliability of the included studies, eligibility criteria were established for both preeclampsia and periodontitis.

• Preeclampsia criteria: Preeclampsia was defined as the onset of hypertension (systolic blood pressure ≥140 mmHg or diastolic blood pressure ≥90 mmHg) after 20 weeks of gestation, accompanied by proteinuria (≥300 mg/24 h) or, in the absence of proteinuria, evidence of systemic involvement, such as thrombocytopenia, renal insufficiency, impaired liver function, pulmonary edema, or cerebral/visual symptoms, according to the guidelines set forth by the American College of Obstetricians and Gynecologists (ACOG).
• Periodontitis criteria: Periodontitis was defined based on clinical parameters, including clinical attachment loss (CAL), pocket probing depth (PPD), bleeding on probing (BOP), and radiographic evidence of alveolar bone loss. Studies that defined PD according to these clinical or radiographic parameters were considered eligible.

### 2.2. Study Execution Method

**Figure 1:**
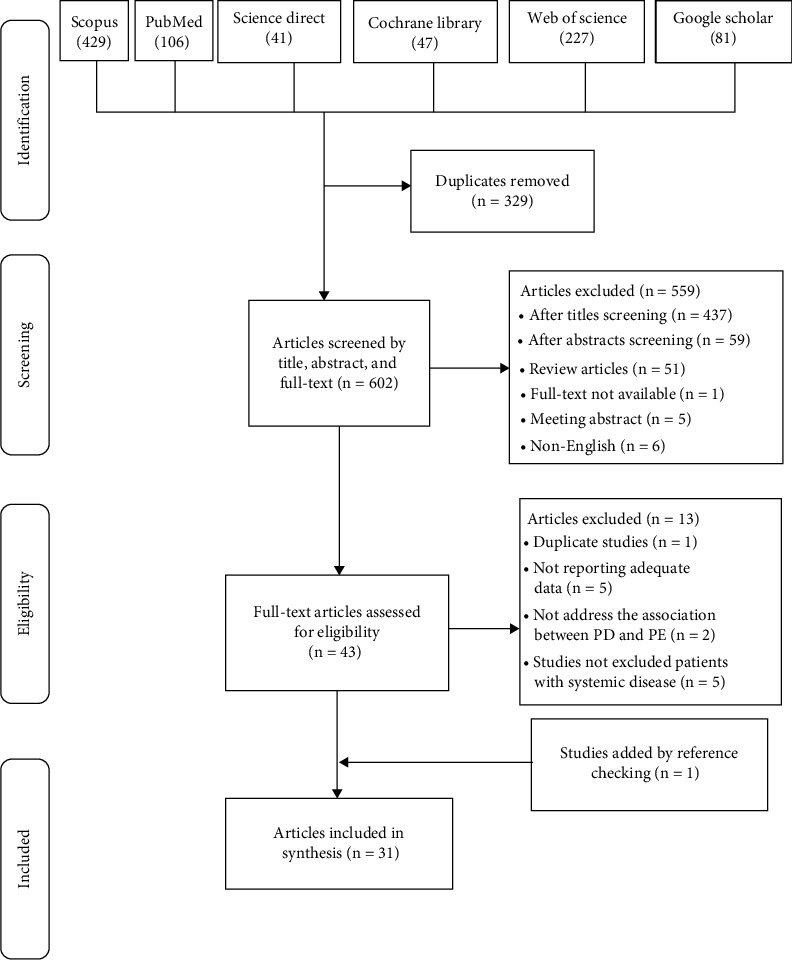
Selection process in the research databases based on PRISMA checklist.

Initially, the main keywords were determined using the PICO model, and preferred terms and their equivalents were identified based on Medical Subject Headings (MeSH). Searches were then conducted in reputable databases. The PRISMA checklist was used to select articles. In the next stage, initial screening of articles was performed based on the inclusion and exclusion criteria, and the resulting articles were subjected to quality evaluation. Quality assessment of articles was conducted using the standard Newcastle Ottawa Scale (NOS Scale) [12]. Finally, articles eligible for detailed review were fully read, and necessary items were extracted based on the data extraction form (Figure 1).

For data collection, search keywords included combinations of terms, such as “PDs” OR “Periodontitis” OR “periodontium” OR “gum disease” AND “pre-eclampsia” OR “preeclampsia” OR “Toxemia of Pregnancy” OR “pregnancy complications” OR “pregnancy hypertension” OR “Pregnancy Toxemias.“

### 2.3. Data Analysis Method

Considering that the main indices examined in this research were the mean and standard deviation, the variance was calculated using the normal distribution, and a 95% confidence interval was computed. To test for heterogeneity among studies, indices, such as *P*, *I*^2^, and *T*^2^ were used. The statistical software STATA 14 was employed for data description and central indices, such as mean, standard deviation, and variance. Additionally, analyses were performed using the random effects model with the Mantel–Haenszel method, and the mean increases in blood pressure and urine protein were reported using a Forest plot.

Table 2 provides an overview of all included studies examining the association between maternal periodontitis and the development of preeclampsia. Across these investigations, definitions of periodontitis varied, ranging from CAL and periodontal pocket depth (PPD) thresholds to composite indices incorporating BOP, plaque index, and gingival index (GI). However, preeclampsia was uniformly defined by elevated blood pressure in the presence of proteinuria (Table 2).

## 3. Results

**Figure 2:**
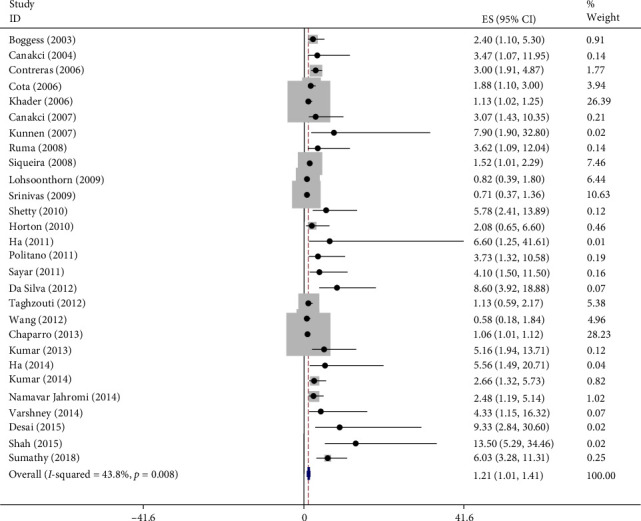
Accumulation chart of the effect of periodontitis on preeclampsia. *Note:* Weights are from random effects analysis.

As illustrated in Figure 2, the random effects meta-analysis yielded a pooled odds ratio (OR) of 1.21 (95% CI 1.01–1.41) for the association between maternal periodontitis and preeclampsia (*I*^2^ = 43.8%) (Figure 2).

### 3.1. Heterogeneity Analysis

Chi-square test: The chi-square test (*χ*^2^ = 48.03, d.f. = 27, *p*=0.008) confirmed the presence of heterogeneity among the included studies, suggesting variability in study results.

Tau-square test: The tau-square test (*τ*^2^ = 0.0372) also indicated heterogeneity in the studies.

However, the *I*^2^ test (43.8%) demonstrated that the level of heterogeneity was moderate. To account for this, a random effects model was applied, and further meta-regression analysis confirmed that heterogeneity was not influenced by publication year, examination time, or OR type.

### 3.2. Meta-Analysis Results

Using the Mantel–Haenszel method under the random effects model, the combined OR was 1.21 (95% CI: 1.01–1.42, *p* < 0.05), indicating a statistically significant association. This suggests that women with periodontitis have a 21% higher risk of developing preeclampsia compared to healthy individuals.

### 3.3. Temporal Analysis

**Figure 3:**
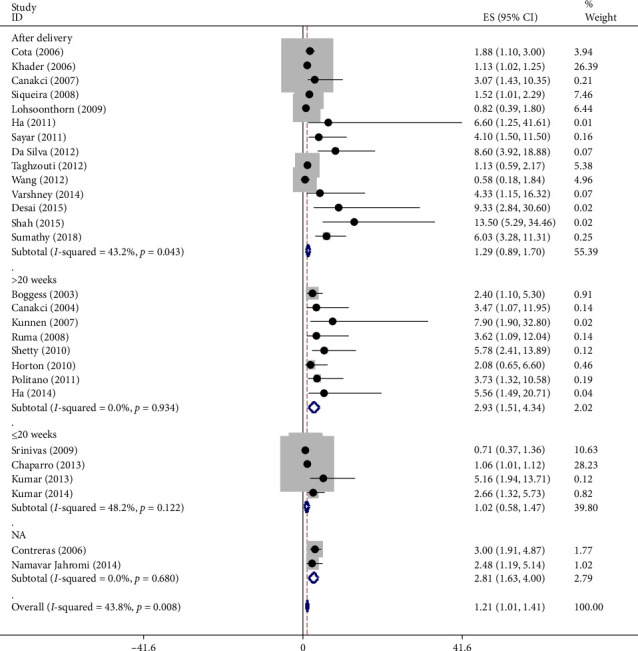
Accumulation chart of sthe effect of periodontitis on preeclampsia based on the time of experiment. *Note:* Weights are from random effects analysis.

The risk of preeclampsia varied based on the timing of periodontal examination as shown in Figure 3:

  Postpartum: The OR was 1.3 suggesting a 30% increased risk.

After 20 weeks of pregnancy: The OR increased to 2.9 indicating a 190% higher risk compared to healthy women.

### 3.4. OR Type Analysis

**Figure 4:**
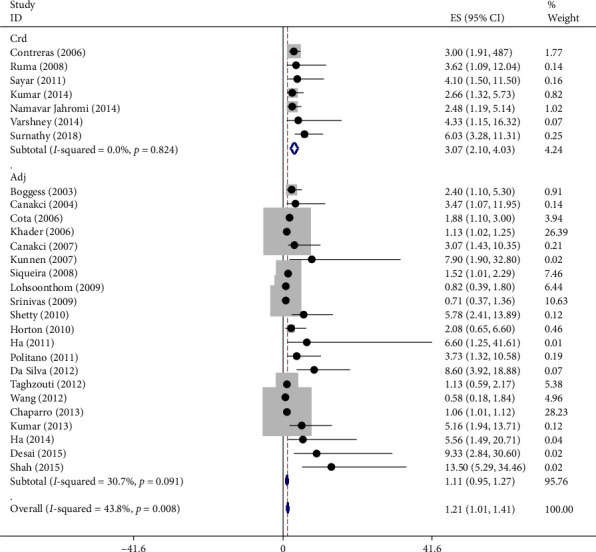
Accumulation chart of the effect of periodontitis on preeclampsia based on odds ratio (OR). *Note:* Weights are from random effects analysis.

As illustrated in Figure 4, when stratified by the type of OR used:

The raw OR indicated that women with periodontitis had twice the risk of developing preeclampsia compared to healthy women.

The adjusted OR was lower, suggesting that controlling for confounding factors may reduce the observed association but does not eliminate it.

### 3.5. Publication Biass

Begg's test: Begg's test indicated no publication bias in the studies reviewed (Table 3).

Meta-regression analysis: Meta-regression showed that variables, such as publication year, examination time, and OR type were not the causes of heterogeneity (Table 4).

## 4. Discussion

### 4.1. Key Findings and Clinical Implications

The results of this study indicate a statistically significant difference in the risk of preeclampsia between periodontal patients and healthy individuals. Specifically, women with periodontitis have a 21% higher chance of developing preeclampsia compared to healthy individuals. This finding underscores the potential role of maternal periodontal health in pregnancy outcomes, suggesting that periodontitis may serve as an important modifiable risk factor in the prevention of preeclampsia.

To further investigate the relationship, a temporal analysis was conducted: The risk of preeclampsia was 1.3 times higher postpartum in women with periodontitis. The risk was 2.9 times higher after 20 weeks of pregnancy, suggesting a stronger association as pregnancy progresses.

These findings indicate that inflammatory processes associated with periodontitis may contribute to the pathophysiology of preeclampsia, particularly in later stages of pregnancy when systemic inflammation plays a more prominent role.

### 4.2. Comparison With Existing Literature

The findings of this meta-analysis are consistent with previous research, indicating a significant correlation between PD and preeclampsia. Le et al. [41] conducted a meta-analysis of six cohort studies and 24 case–control studies, concluding that PD significantly increases the risk of preeclampsia, particularly in low- to middle-income countries. Similarly, Wei et al. [10] reviewed 13 case–control studies and two cohort studies, finding a 2.79-fold increased risk of preeclampsia in women with PD.

In another review by Konopka and Zakrzewska [42], cohort studies clearly indicated an increased risk of preeclampsia in patients with periodontitis. Additionally, reviews by Daalderop et al. [43], Matevosyan [44], and Sgolastra et al. [45] also concluded that maternal chronic periodontitis is an independent predictor of preeclampsia–. Sumathy et al. 40] and Desai et al. [38] demonstrated that maternal periodontitis is associated with an increased risk of preeclampsia. Jaiman et al. [46] showed that PD is more prevalent in pregnant women with preeclampsia than those with normal blood pressure. These studies support the hypothesis that periodontal tissue destruction is associated with an increased risk of preeclampsia, as observed in this meta-analysis.

Contrarily, Huang et al. [11] conducted a meta-analysis of 11 observational studies and found no significant association between PD and preeclampsia. This discrepancy could be attributed to variations in study populations, PD severity, and individual and environmental factors.

Also, studies by Rezavand et al. [47] found no significant statistical relationship between preeclampsia and PD parameters in pregnant women. Similarly, Khader et al. [17] and Taghzouti et al. [30] found no significant statistical relationship between severe periodontitis and increased preeclampsia risk, contrasting with our meta-analysis results. It's important to note that these studies were not meta-analyses and differed in nature from our study.

### 4.3. Biological Mechanisms Linking Periodontitis and Preeclampsia

The association between periodontitis and preeclampsia can be explained through several biological pathways:

Inflammation and systemic immune response: Periodontal infections induce chronic systemic inflammation, increasing the production of pro-inflammatory cytokines (IL-6 and TNF-*α*) and CRP, which are also implicated in preeclampsia [26].

Oxidative stress and endothelial dysfunction: Both periodontitis and preeclampsia are associated with increased oxidative stress, which leads to endothelial dysfunction—a key mechanism in preeclampsia pathogenesis [26].

Bacterial translocation and placental dysfunction: Periodontal pathogens, such as *P. gingivalis* can enter the bloodstream, potentially affecting placental development and function, contributing to hypertension and preeclampsia [26].

### 4.4. Clinical and Public Health Implications

From a clinical perspective, the prevention of preeclampsia through the management of PD is a plausible strategy. Reducing the incidence of preeclampsia can be achieved by addressing its risk factors, including PD. Despite evidence from clinical trials suggesting that periodontal treatment during pregnancy does not significantly reduce the risk of preeclampsia, it is clear that maintaining periodontal health is crucial for overall maternal health.

Bi et al. [48] demonstrated that while periodontal treatment during pregnancy significantly reduces neonatal mortality, it does not have a statistically significant effect on the incidence of preeclampsia, gestational diabetes, cesarean delivery, or low birth weight. This suggests that other mechanisms and factors may be involved in the development of preeclampsia, and further research is needed to elucidate these pathways [48].

Govindasamy et al. [49] conducted a systematic review showing that nonsurgical periodontal treatment during pregnancy is safe and significantly reduces adverse pregnancy outcomes in high-risk patients. Therefore, it can be considered part of prenatal care, and maternal PD should be recognized as an independent risk factor for adverse pregnancy outcomes. Consequently, periodontal treatment is essential for improving maternal health and should be recommended by qualified individuals before pregnancy.

Several mechanisms may explain the relationship between PD and preeclampsia. Periodontal pathogens can induce systemic inflammation and oxidative stress, potentially affecting placental function and leading to preeclampsia [50]. Additionally, both conditions share common risk factors, such as socioeconomic status and genetic predisposition, which could contribute to their co-occurrence [50].

Moreover, our study found a statistically significant difference in preeclampsia risk at different testing times between the two groups. The OR for preeclampsia in the periodontitis group was 1.3 times higher postpartum and 2.9 times higher during pregnancy beyond 20 weeks compared to the healthy group. Overall, the OR for preeclampsia in the periodontitis group was twice that of the healthy group.

Given the significant relationship between periodontitis and preeclampsia found in this meta-analysis, it is crucial for obstetricians to pay attention to the oral hygiene of their patients and timely refer them to dentists. In this regard, Nguyen et al. [51] demonstrated that midwives play a key role in educating pregnant women about oral hygiene and facilitating their access to necessary dental care. Therefore, screening and preventing PD before pregnancy should be included in maternal health care programs to improve oral health and pregnancy outcomes [52].

Considering the results of this meta-analysis and the prevalence of PD in society, and given that young women and pregnant mothers are at risk for this disease, awareness and information dissemination through interdisciplinary cooperation, including among obstetricians, dental staff, and midwives, are crucial for achieving optimal oral health and improving pregnancy outcomes. Moreover, accurate health education for women and encouraging adherence, especially during pregnancy, and educating dental staff on specific pregnancy-related issues can be beneficial and effective.

The limitations of this study include the inability to standardize the studies in terms of race, economic status, and nutritional status, which are known to influence the prevalence and severity of PD. Additionally, variations in healthcare systems and preventive measures across different countries may affect the incidence of PD and preeclampsia, leading to heterogeneity in the results.

## 5. Conclusion

This meta-analysis provides compelling evidence that PD is associated with an increased risk of preeclampsia. Given the significant public health implications, it is crucial for healthcare providers to recognize the importance of oral health in pregnant women. Integrating periodontal care into routine prenatal care could potentially reduce the incidence of preeclampsia and improve overall pregnancy outcomes. Future research should focus on elucidating the underlying mechanisms and developing standardized protocols for the assessment and management of PD in pregnant women.

Given the high prevalence of PD in the general population and the potential for serious pregnancy complications, it is imperative to enhance awareness and education regarding oral health during pregnancy. Collaboration between obstetricians, dental professionals, and midwives is essential to ensure comprehensive care for pregnant women, ultimately improving maternal and fetal health outcomes.

## Figures and Tables

**Table 1:** Inclusion and exclusion criteria.

Inclusion criteria	Exclusion criteria
Articles published in English.	Articles involving patients with systemic diseases.
Articles that were one of the types of cross-sectional, cohort, or case–control studies.	Articles that did not control any confounding variables.
Articles that analyzed the association between periodontal disease and preeclampsia.	Articles containing duplicate information.
Articles that provided a clear definition of periodontal disease and preeclampsia.	Articles lacking sufficient information.
Articles that described periodontal disease using clinical methods or radiographic parameters.	Review articles and letters to the editor.
Articles focused on human samples.	—

**Table 2:** Summary of included studies.

Study	Definition of periodontitis	Definition of preeclampsia	Key findings
Boggess et al. [13]	Clinical attachment loss (CAL) ≥3 mm at ≥3 sites	Blood pressure ≥140/90 mmHg with proteinuria	OR = 2.40 (1.10, 5.30)
Canakci et al. [14]	Periodontal pocket depth (PPD) >4 mm, CAL ≥ 3 mm	Hypertension with proteinuria	OR = 3.47 (1.07, 11.95)
Contreras et al. [15]	Periodontal Index (PI) >2	BP ≥140/90 mmHg with proteinuria	OR = 3.00 (1.91, 4.87)
Cota et al. [16]	PPD > 4 mm, CAL ≥ 3 mm, BOP	BP ≥140/90 mmHg, proteinuria	OR = 1.88 (1.10, 3.00)
Khader et al. [17]	PPD ≥ 4 mm, CAL ≥ 3 mm	BP ≥140/90 mmHg, proteinuria	OR = 1.13 (1.02, 1.25)
Canakci et al. [18]	PI and gingival index (GI)	BP ≥140/90 mmHg, proteinuria	OR = 3.07 (1.43, 10.35)
Kunnen et al. [19]	CAL ≥ 3 mm, PPD ≥ 4 mm	BP ≥140/90 mmHg, proteinuria	OR = 7.90 (1.90, 32.80)
Ruma et al. [20]	PPD ≥ 4 mm, GI score	BP ≥140/90 mmHg, proteinuria	OR = 3.62 (1.09, 12.04)
Siqueira et al. [21]	CAL ≥ 3 mm, PI score	BP ≥140/90 mmHg, proteinuria	OR = 1.52 (1.01, 2.29)
Lohsoonthorn et al. [22]	PPD ≥ 4 mm, BOP	BP ≥140/90 mmHg, proteinuria	OR = 0.82 (0.39, 1.80)
Srinivas et al. [23]	CAL ≥ 3 mm, radiographic bone loss	BP ≥140/90 mmHg, proteinuria	OR = 0.71 (0.37, 1.36)
Shetty et al. [24]	PI, BOP, PPD ≥ 4 mm	BP ≥140/90 mmHg, proteinuria	OR = 5.78 (2.41, 13.89)
Horton et al. [25]	CAL ≥ 3 mm, PPD ≥ 4 mm	BP ≥140/90 mmHg, proteinuria	OR = 2.08 (0.65, 6.60)
Ha et al. [26]	Radiographic bone loss, CAL ≥ 3 mm	BP ≥140/90 mmHg, proteinuria	OR = 6.60 (1.25, 41.61)
Politano et al. [27]	PPD ≥ 4 mm, BOP	BP ≥140/90 mmHg, proteinuria	OR = 3.73 (1.32, 10.58)
Sayar et al. [28]	PI, CAL ≥ 3 mm, GI score	BP ≥140/90 mmHg, proteinuria	OR = 4.10 (1.50, 11.50)
Moura Da Silva et al. [29]	PPD ≥ 4 mm, BOP, PI	BP ≥140/90 mmHg, proteinuria	OR = 8.60 (3.92, 18.38)
Taghzouti et al. [30]	CAL ≥ 3 mm, BOP	BP ≥140/90 mmHg, proteinuria	OR = 1.30 (0.59, 2.17)
Wang et al. [31]	PI, BOP, CAL ≥ 3 mm	BP ≥140/90 mmHg, proteinuria	OR = 0.58 (0.18, 1.84)
Chaparro et al. [32]	PPD ≥ 4 mm, BOP	BP ≥140/90 mmHg, proteinuria	OR = 1.06 (1.01, 1.12)
Kumar et al. [33]	PI, CAL ≥ 3 mm, radiographic bone loss	BP ≥140/90 mmHg, proteinuria	OR = 5.16 (1.94, 13.71)
Ha et al. [34]	BOP, CAL ≥ 3 mm	BP ≥140/90 mmHg, proteinuria	OR = 5.56 (1.49, 20.71)
Kumar et al. [35]	PI, PPD ≥ 4 mm	BP ≥140/90 mmHg, proteinuria	OR = 2.66 (1.32, 5.73)
Namavar Jahromi et al. [36]	GI, CAL ≥ 3 mm	BP ≥140/90 mmHg, proteinuria	OR = 2.48 (1.19, 5.14)
Varshney and Gautam [37]	PPD ≥ 4 mm, radiographic bone loss	BP ≥ 140/90 mmHg, proteinuria	OR = 4.33 (1.15, 16.32)
Desai et al. [38]	CAL ≥ 3 mm, BOP	BP ≥140/90 mmHg, proteinuria	OR = 9.33 (2.84, 30.60)
Shah et al. [39]	PI, GI, BOP, PPD ≥ 4 mm	BP ≥140/90 mmHg, proteinuria	OR = 13.50 (5.29, 34.46)
Sumathyet al. [40]	Radiographic bone loss, PI	BP ≥140/90 mmHg, proteinuria	OR = 6.03 (3.28, 11.31)

**Table 3:** Tests for publication bias.

Odds ratio type	Number	Begg's		Begg's	
		Score	Standard deviation	*z*	*p*
Adjusted	21	52	33.116	1.57	0.116
Raw	7	3	6.658	0.45	0.652
Overall	28	55	33.779	1.63	0.103

**Table 4:** Meta-regression analysis.

• db metareg						
• metareg logOR Publicationyear ExaminationTime ORtype, wsse (selogOR) bsest (reml)						
Meta-regression				Number of obs = 26		
REML estimate of between-study variance				Tau2 = 0.02087		
% residual variation due to heterogeneity				*I*-squared_res = 0.00%		
Proportion of between-study variance explained				Adj *R*-squared = 52.39%		
Joint test for all covariates				Model *F* (3, 22) = 2.11		
With Knapp–Hartung modification				Prob > *F* = 0.1286		

**LogOR**	**Coef.**	**Std. err.**	***T***	***p* > |t|**	**(95% Conf. interval)**	

Publication year	0.0331892	0.0239962	1.38	0.181	−0.0165758	0.0829543
Examination time	0.1368044	0.0883458	1.55	0.136	−0.0464136	0.3200224
OR type	−0.2355082	0.2542958	−0.93	0.364	−0.7628854	0.291869
_cons	−66.31994	48.55975	−1.37	0.186	−167.0267	34.38681

## Data Availability

The data that support the findings of this study are available from the corresponding author upon reasonable request.
